# Growth Differentiation Factor-15 Produces Analgesia by Inhibiting Tetrodotoxin-Resistant Nav1.8 Sodium Channel Activity in Rat Primary Sensory Neurons

**DOI:** 10.1007/s12264-021-00709-5

**Published:** 2021-06-02

**Authors:** Wei Lin, Wen-Wen Zhang, Ning Lyu, Hong Cao, Wen-Dong Xu, Yu-Qiu Zhang

**Affiliations:** 1 Department of Translational Neuroscience, Jing’an District Centre Hospital of Shanghai, State Key Laboratory of Medical Neurobiology and Institutes of Brain Science, Fudan University, Shanghai, 200032 China; 2 Department of Hand Surgery, Huashan Hospital, Fudan University, Shanghai, 200040 China; 3 Department of Neurobiology, School of Basic Medical Sciences, Shanghai Medical College, Fudan University, Shanghai, 200032 China

**Keywords:** Growth differentiation factor-15, Tetrodotoxin-resistant sodium channel Nav1.8, Dorsal root ganglion, Whole-cell recording, Activin receptor-like kinase-2, Pain

## Abstract

Growth differentiation factor 15 (GDF-15) is a member of the transforming growth factor-β superfamily. It is widely distributed in the central and peripheral nervous systems. Whether and how GDF-15 modulates nociceptive signaling remains unclear. Behaviorally, we found that peripheral GDF-15 significantly elevated nociceptive response thresholds to mechanical and thermal stimuli in naïve and arthritic rats. Electrophysiologically, we demonstrated that GDF-15 decreased the excitability of small-diameter dorsal root ganglia (DRG) neurons. Furthermore, GDF-15 concentration-dependently suppressed tetrodotoxin-resistant sodium channel Nav1.8 currents, and shifted the steady-state inactivation curves of Nav1.8 in a hyperpolarizing direction. GDF-15 also reduced window currents and slowed down the recovery rate of Nav1.8 channels, suggesting that GDF-15 accelerated inactivation and slowed recovery of the channel. Immunohistochemistry results showed that activin receptor-like kinase-2 (ALK2) was widely expressed in DRG medium- and small-diameter neurons, and some of them were Nav1.8-positive. Blockade of ALK2 prevented the GDF-15-induced inhibition of Nav1.8 currents and nociceptive behaviors. Inhibition of PKA and ERK, but not PKC, blocked the inhibitory effect of GDF-15 on Nav1.8 currents. These results suggest a functional link between GDF-15 and Nav1.8 in DRG neurons *via* ALK2 receptors and PKA associated with MEK/ERK, which mediate the peripheral analgesia of GDF-15.

**Supplementary Information:**

The online version contains supplementary material available at 10.1007/s12264-021-00709-5.

## Introduction

Growth differentiation factor-15 (GDF-15), also known as macrophage inhibitory cytokine-1, is a novel member of the bone morphogenetic protein (BMP)/transforming growth factor-β (TGF-β) superfamily, which is widely distributed in the central and peripheral nervous systems [1]. It has been shown to play multiple roles in various physiological and pathological processes including growth differentiation, neuroprotection, inflammation, cancer, tissue injury, repair, and nerve regeneration [2–5]. In the central nervous system (CNS), GDF-15 protects nigrostriatal dopaminergic neurons in the 6-hydroxydopamine-lesioned model and reduces losses of dopaminergic neurons [6, 7]. In the periphery, GDF-15 maintains the survival of postnatal dorsal root ganglia (DRG) neurons and spinal, facial, as well as trigeminal motoneurons [8]. After peripheral nerve injury (e.g. mouse optic nerve or sciatic nerve injury), GDF-15 mRNA and protein are upregulated [4, 9] and local application of GDF-15 into the lesioned sciatic nerve promotes sensory regeneration [4]. As an anti-inflammatory cytokine, GDF-15 has also been shown to have anti-inflammatory effects [10–15]. Despite the fact that GDF-15 has been implicated in peripheral nerve injury and inflammation, it is unclear whether it regulates nociceptive transmission.

Several members of the BMP/TGF-β superfamily, such as Activins, BMPs, and TGF-βs, are involved in the modulation of pain signaling [16–19]. A previous study from our lab showed that peripheral TGF-β1 signaling contributes to bone cancer pain *via* regulating transient receptor potential vanilloid-1 (TRPV1) in primary sensory neurons [20]. In this study, we further investigated whether and how GDF-15 modulates peripheral nociceptive signaling in DRG neurons.

The effects on DRG ion channels may be an important mechanism of peripheral nociceptive modulation [21–24]. In neuropathic and inflammatory pain conditions, voltage-gated Na^+^ channels in nociceptive primary sensory neurons are involved in the development of peripheral hyperexcitability [25, 26]. Among them, the tetrodotoxin-resistant (TTX-R) Na^+^ channel Nav1.8, mainly expressed by small- and medium-diameter DRG neurons [27, 28], substantially contributes to the upstroke of the action potential (AP) [29, 30]. Nav1.8-null mice show an increased threshold to noxious mechanical and thermal stimuli as well as delayed development of inflammatory pain [31]. Functional knockdown of Nav1.8 reduces the nociceptive hypersensitivity in neuropathic pain and inflammatory pain models [25, 32, 33]. In patients with painful small-fiber neuropathy, several Nav1.8 mutations have been identified, and some of these Nav1.8 mutations enhance the channel’s response to depolarization and increase the excitability of DRG neurons [29, 34]. As an important target of pain relief, Nav1.8 is modulated by multiple drugs and molecules including pro- and anti-inflammatory cytokines [35–37].

GDF-15 has been reported to increases the outward K^+^ and Ca^2+^ currents in rat cerebellar granule neurons by the Smad-independent Akt/mTOR and ERK signaling pathways [38, 39]. Several second-messenger cascades including ERK, PKA, and PKC have been shown to regulate Nav1.8 channels [25, 40]. It is reasonable to hypothesize that GDF-15 may participate in pain modulation by regulating Nav1.8 channels to change DRG nociceptive neuronal excitability.

## Materials and Methods

### Animals

Adult female Wistar rats (120–180 g) were purchased from Shanghai Experimental Animal Center of the Chinese Academy of Sciences. The rats were housed under a 12/12 h light/dark cycle with a room temperature (RT) of 22 ± 1°C and received food and water *ad libitum*. All experimental procedures were approved by the Committee on the Use of Animal Experiments of Fudan University (permit No. SYXK 2009–0082) and followed the policies on the use of laboratory animals issued by the International Association for the Study of Pain (Washington D.C.). After the experiments, the rats were sacrificed by carbon dioxide inhalation. All the experiments including behavioral tests, electrophysiological recordings, and immunohistochemistry were performed by experimenters who were blinded to the treatments.

### Drugs and Chemicals

GDF-15 was from Pepro Tech (120–28, Rocky Hill, NJ, USA). DMH-1, a selective ALK-2 receptor inhibitor, was from Selleck (S7146, Houston, TX, USA). All the other drugs were from Sigma-Aldrich (St. Louis, MO, USA). GDF-15 was dissolved in sterile 0.01 mol/L PBS with 0.1% BSA and the other drugs were dissolved in normal saline. The drug dosages were selected based on our preliminary studies and previous reports. GDF-15 was applied to the chamber for 10–30 min.

### Preparation of DRG Neurons and Patch-Clamp Recordings

Acute isolation of DRG neurons was as described previously [20]. Briefly, rats were anesthetized with isoflurane and then rapidly decapitated. The L3–L6 DRGs were removed and immediately transferred to Dulbecco’s modified Eagle’s medium (DMEM; Invitrogen, Thermo Fisher Scientific, Carlsbad, CA, USA) on ice. The ganglia were minced and treated with collagenase (type IA, 2.67 mg/mL, Millipore Sigma, Billerica, MA, USA) and trypsin (type I, 1 mg/mL, Millipore Sigma) in DMEM saturated with CO_2_/O_2_ mixed gas at 37°C for 30 min. For electrophysiological recording, the isolated DRG neurons were plated onto glass coverslips in culture dishes and incubated with a standard external solution.

Whole-cell recordings were performed in DRG neurons with an Axonpatch 200B amplifier (Axon Instruments, USA) as described previously [20, 40]. Recording electrodes (ID. 0.86 mm, OD. 1.5 mm, BF 150-86-10, Sutter Instruments, USA) were pulled on a P-97 puller (Sutter instruments, USA) with a resistance of 3–5 MΩ. The pipette solution contained (in mmol/L): 140 KCl, 1 MgCl_2_, 0.5 CaCl_2_, 5 EGTA, 10 HEPES, 3 Na_2_ATP, 0.2 NaGTP, pH was adjusted to 7.2 with KOH. Seals (>1 GΩ) were established between the electrode and the cells. The cell membrane capacitance and series resistance were compensated (>80%) after the whole-cell configuration was established. An online p/4 protocol was used for leak currents. Signals were filtered at 2 kHz and sampled at 10 kHz. For Nav1.8 current recordings, the external solution contained (in mmol/L): 32 NaCl, 20 TEA-Cl, 105 choline chloride, 1 MgCl_2_, 1 CaCl_2_, 1 CdCl_2_, 10 HEPES, 10 glucose, and 0.0005 TTX, adjusted to pH 7.4 with NaOH. The pipette solution contained (in mmol/L): 140 CsF, 1 MgCl_2_, 1 EGTA, 5 Na_2_ATP, 10 HEPES, pH was adjusted to 7.2 with CsOH. DRG neurons were held at –60 mV and Nav1.8 currents were elicited by depolarizing pulses to –10 mV. The activation and inactivation properties of Nav1.8 currents were recorded with the appropriate voltage protocols. The voltage-clamp protocol (50 ms depolarizing steps from –50 mV to +5 mV at 5-mV increments) was used to determine the activation of Nav1.8 channels. The Boltzmann function of the form *G*_Na_/*G*_Namax_* =* 1/{1 *+ exp* [(*V*_m1/2_ *− V*_m_)/*k*]} was used to describe the voltage dependence of activation and half-activation potential. Steady-state inactivation of the Nav1.8 channel was determined at a series of membrane potentials from –60 mV to –5 mV at 5-mV increments for 100 ms and a following –10 mV test potential. The Boltzmann function *I*_Na_/*I*_Namax_* =* 1/{1 *+ exp* [(*V* – *V*_m1/2_)/*k*]} was used to describe the steady-state inactivation curve, in which *I*_Namax_ is the maximal peak current and *V* is the prepulse membrane potential. All of the recordings were performed in small-diameter (<25 μm) DRG neurons. pClamp 9.0 (Molecular Devices, Foster City, CA, USA) software was used during experiments and analysis.

### Immunohistochemistry

Rats were deeply anesthetized with an overdose of urethane (1.5 g/kg) and perfused with normal saline followed by 4% cold paraformaldehyde. L3–L6 DRGs were removed and postfixed in the same fixative for 4–6 h, and then immersed in a gradient of sucrose (10%, 20%, and 30%) for 24–48 h at 4°C for cryoprotection. DRG sections (14 μm) were cut on a Cryostat (CM1950, Leica, Wetzlar, Germany), mounted on silicone-wrapped slides, and stored at –80°C until immunofluorescence labeling. Sections were incubated in blocking solution (10% normal donkey serum in 0.01 mol/L PBS with 0.3% Triton X-100) for 2 h at RT, then overnight at 4°C with the following primary antibodies: goat anti-ALK2 (1:100, R&D Systems, AF637, Minneapolis, MN, USA), rabbit anti-Nav1.8 (1:200, ASC-016, Alomone, Jerusalem, Israel), rabbit anti-substance P (1:4000, Peninsula Labs, RIN7451, San Carlos, CA, USA), rabbit anti-CGRP, 1:20000, Peninsula Labs, IHC6006), mouse anti-peripherin (1:1000, Millipore, mab1527, Billerica, MA, USA). The sections were incubated with a mixture of Alexa Fluor 488- or Alexa Fluor 546-conjugated secondary antibodies (1:200, Invitrogen, A11055/A11036, Thermo Fisher Scientific, Waltham, MA, USA), or IB4-Alexa Fluor 488 (1:1000, Invitrogen, A21206, Thermo Fisher Scientific) for 2 h at RT. Omitting the primary antibodies and pre-absorption experiments were used to verify the specificity of immunostaining and primary antibodies. The stained sections were examined under a confocal laser-scanning microscope (FV1000, Olympus, Tokyo, Japan).

### Behavioral Experiments

#### von Frey Test for Mechanical Pain

Rats were acclimated to the testing environment for 2–3 days before testing. Paw withdrawal thresholds (PWTs) in response to *von* Frey hairs (1–26 g, Stoelting Co., Wood Dale, IL, USA) were measured to determine the mechanical stimulation response threshold. Each rat was placed in a Plexiglas box (10 × 20 × 20 cm^3^) on an elevated metal mesh floor and habituated for 30 min. A series of *von* Frey hair stimuli (1.0, 1.4, 2, 4, 6, 8, 10, 15, and 26 g) were delivered to the plantar surface of the central region of the hind paw. Each hair was applied 5 times at 15-s intervals and each time maintained for 2 s. When the hind paw was withdrawal from a particular hair 3 out of the 5 consecutive applications, the value of the hair in grams was considered the PWT of the rat.

#### Hargreaves Test for Thermal Pain

The testing environment was the same as for the *von* Frey test. Paw withdrawal latencies (PWLs) in response to a radiant heat stimulus were measured to evaluate the thermal response threshold. Rats were placed in a Plexiglas box (10 × 20 × 20 cm^3^) on an elevated glass platform and acclimatized for 30 min before testing. A radiant heat source (IITC Life Science Instruments, Woodland Hills, CA, USA) was turned off when the rat lifted its foot. The time from the onset of radiant heat application to the withdrawal of the hind paw was defined as the PWL. The cut-off valve was set to 20 s to preventing tissue damage in the absence of a response.

### Induction of Monoarthritis

Complete Freund’s adjuvant (CFA, 50 μL) was injected into the left ankle articular cavity to induce monoarthritis. The rat was briefly anesthetized with isoflurane and skin around the ankle was sterilized. A 30-gauge needle was inserted vertically to penetrate the skin and turned distally to insert into the articular cavity from the gap between the tibiofibular and tarsus bones until a distinct loss of resistance was felt. Sham arthritic rats were similarly injected with an equal volume of sterile normal saline.

### Statistical Analysis

Data are presented as the mean ± SEM and statistical analyses were performed using GraphPad Prism 7.0 software (San Diego, CA, USA). Statistical comparisons used Student’s *t*-test (comparing 2 groups) or one-way or two-way RM ANOVA followed by the *post hoc* Student-Newman-Keuls test (comparing more than 2 groups). All the hypothesis testing was 2-tailed with *P* <0.05 considered statistically significant.

## Results

### GDF-15 Decreases the Excitability of Nociceptive Primary Sensory Neurons and Suppresses Peripheral Nociceptive Behaviors

**Fig. 1:**
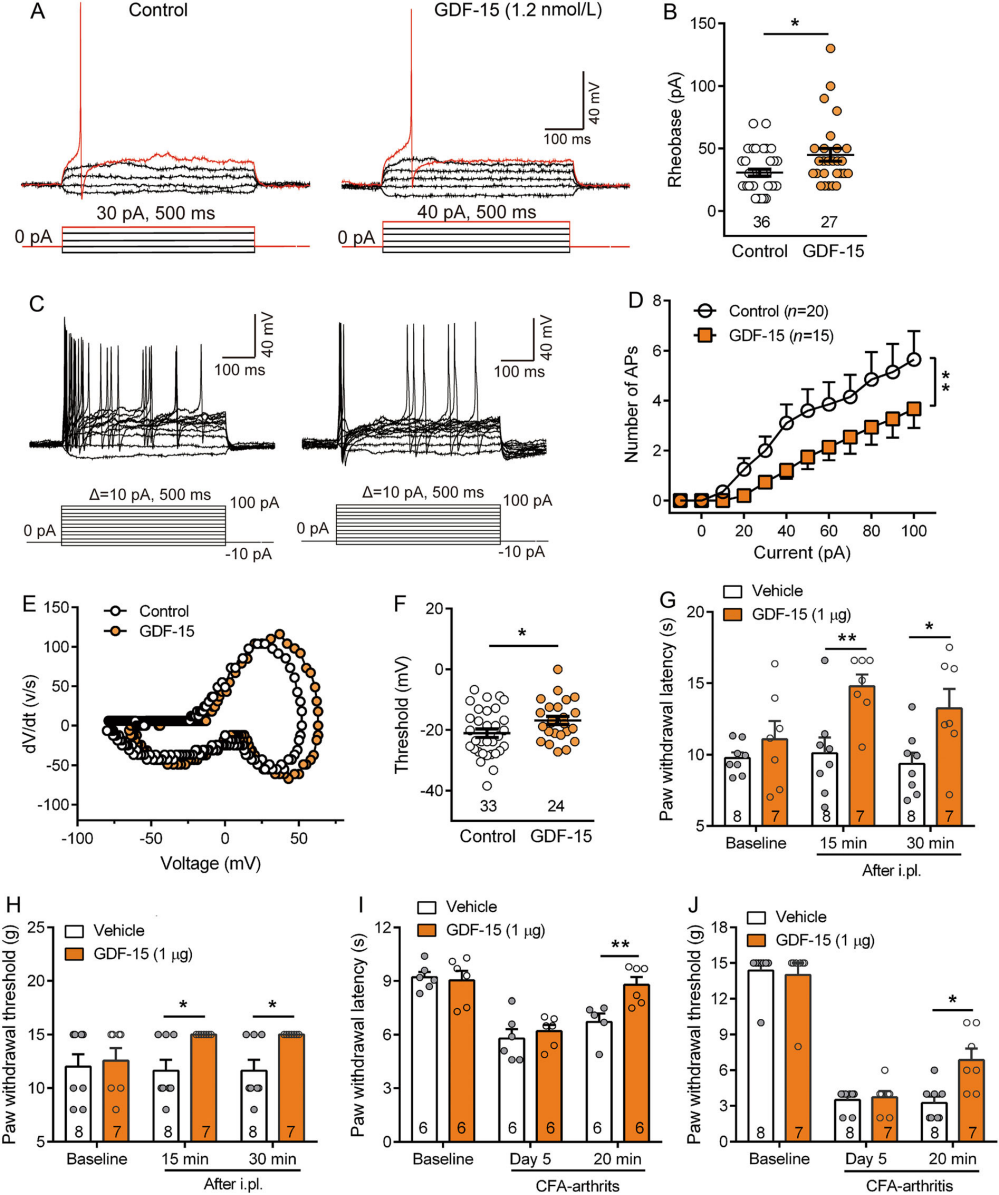
GDF-15 decreases the excitability of nociceptive primary sensory neurons and inhibits nociceptive behaviors. **A.** In current-clamp model, the depolarizing current pulse requires to evoke an action potential (AP) in control and GDF-15 (1.2 nmol/L)-treated small-diameter DRG neurons. **B.** GDF-15 reduces the amount of current required to evoke an AP (*P < 0.05, Student’s *t*-test). **C.** Examples of the AP responses to depolarizing current steps recorded from control and GDF-15 (1.2 nmol/L)-treated small-diameter DRG neurons. **D.** GDF-15 decreases the number of AP discharges in response to 100 pA, 500 ms current injection (**P <0.01, two-way RM ANOVA). **E.** Phase plots of the APs from control and GDF-15 (1.2 nmol/L)-treated small-diameter DRG neurons. **F.** GDF-15 elevates the AP thresholds of small-diameter DRG neurons (*P <0.05, Student’s *t*-test). **G–J.** Intraplantar injection of GDF-15 (1 μg) increased the paw withdrawal latencies to noxious thermal stimulation (**G** and **I**) and paw withdrawal thresholds to *von* Frey mechanical stimulation (**H** and **J**) in naïve (**G** and **H**) and CFA-arthritic rats (**I** and **J**) (*P <0.05; **P <0.01, two-way RM ANOVA).

Previously, we investigated the effects of TGF-β1 on the TRPV1 channel and neuronal excitability in primary sensory neurons, and its role in advanced bone cancer pain [20]. In this study, we further examined the effects of GDF-15, a novel member of the BMP/TGFβ superfamily, on the excitability of DRG neurons and peripheral nociceptive responses. Whole-cell current-clamp recordings showed that bath application of GDF-15 (1.2 nmol/L) significantly increased the rheobase from 30.56 ± 2.76 pA to 44.81 ± 5.16 pA after exposure to GDF-15 (Fig. 1A, B, Student’s *t*-test, *t*_(61)_ = 2.6, *P* = 0.012). In addition, GDF-15 decreased the numbers of APs in response to 100 pA, 500 ms current injection (two-way repeated measures RM ANOVA, treatment: *F*_(1,33)_ = 20.74, *P* <0.0001; Fig 1C, D, S1A, B). Using 500-ms ramp current stimulation from 0 to 100 pA (Δ = 0.2 pA/ms), the latency to the first AP was prolonged and the numbers of APs were reduced (Fig. S1C–E). These data indicated that GDF-15 decreased the excitability of small-diameter DRG neurons. We also examined the effects of GDF-15 on the intrinsic membrane properties of DRG neurons. Analysis of the single AP evoked by step depolarization current stimulation showed that GDF-15-treated neurons had a more depolarized phase-plot curve and AP threshold than vehicle-treated neurons (Fig. 1E, F). No significant differences in resting membrane potential, AP amplitude, half-width, and after-hyperpolarization potential were identified between GDF-15 and control groups (Fig. S1F–I).

The inhibitory effect of GDF-15 on the excitability of small-diameter DRG neurons suggests that peripheral GDF-15 is involved in nociception. Behavioral tests showed that intraplantar injection of GDF-15 (1 μg) significantly inhibited the responses of rats to noxious thermal and *von* Frey mechanical stimuli. The analgesic effect was detected at 15 min after GDF-15 injection (Fig. 1G, H, two-way RM ANOVA, PWL: *F*_(1,13)_ = 16.54, *P* = 0.0002; PWT: *F*_(1,13)_ = 10.43, *P* = 0.003). Considering that previous studies have shown the involvement of GDF-15 in arthritis in both humans and rodents [41, 42], we also examined the effect of GDF-15 on CFA-induced arthritic pain. Intra-ankle articular injection of CFA caused joint edema, erythema, and behavioral hypersensitivity (Fig. S2A–C). Intraplantar injection of GDF-15 inhibited the arthritis-induced thermal hyperalgesia and mechanical allodynia (Fig. 1, J, two-way RM ANOVA, PWL: *F*_(1,10)_ = 4.47, *P* = 0.04; PWT: *F*_(1,13)_ = 4.25, *P* = 0.046). Given that GDF-15 elevated the basal pain response thresholds in normal rats, we further examined the effect of blocking endogenous GDF-15 by neutralizing antibody on nociceptive responses. Intraplantar injection of GDF-15 antibody (GDF-15 Ab, 25 µg) produced mechanical allodynia and thermal hyperalgesia at 0.5 and 1 h, suggesting that GDF-15 also acts as an endogenous analgesic molecule (Fig. S2D, E).

### GDF-15 Suppresses Nav1.8 Currents in Small-Diameter DRG Neurons

**Fig. 2:**
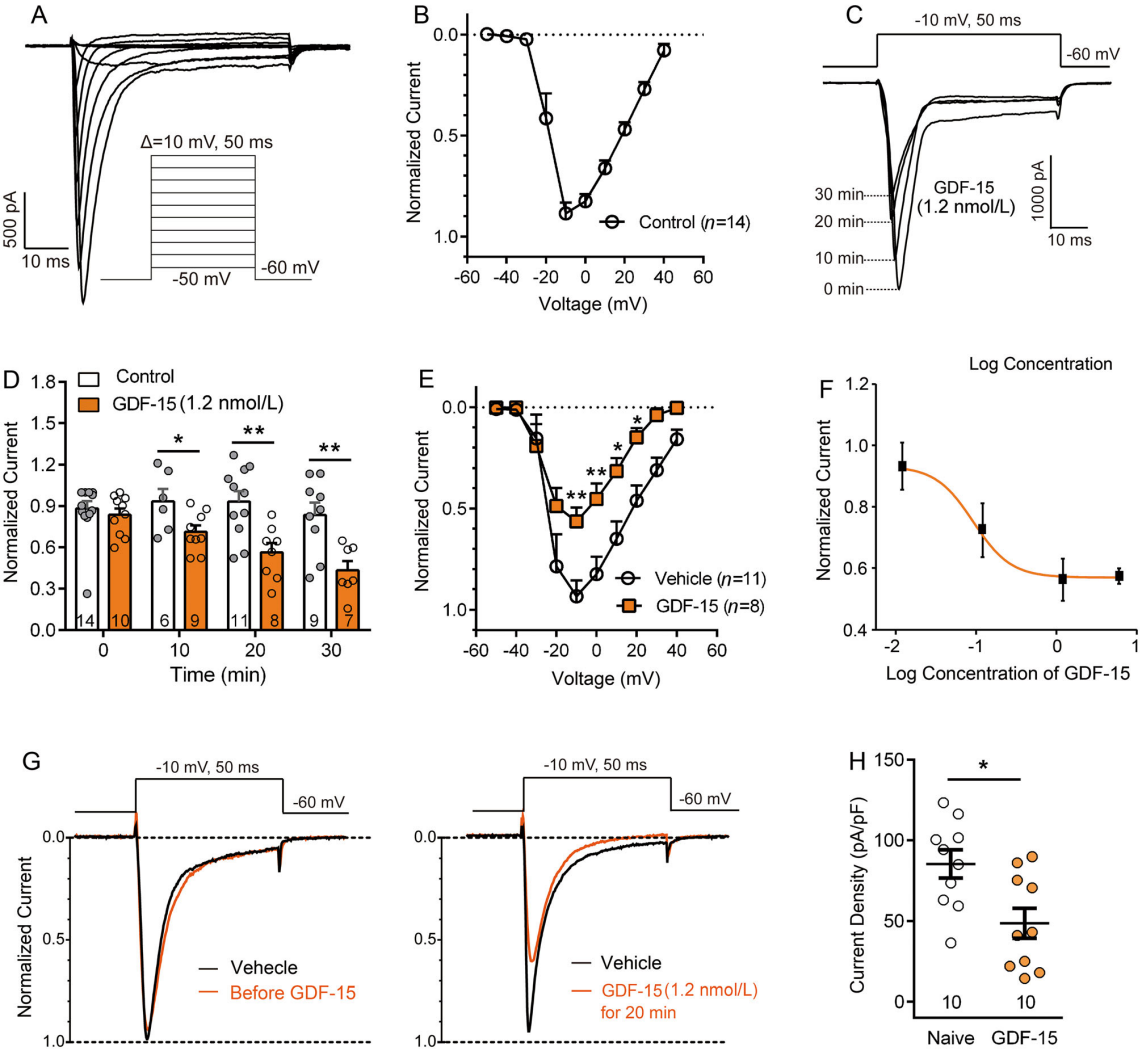
GDF-15 inhibits Nav1.8 currents in small-diameter DRG neurons. **A.** Isolation of TTX-resistant Nav1.8 currents in the presence of 500 nmol/L TTX. Cells were depolarized to a variety of potentials (–50 mV to +40 mV) from a holding potential of –60 mV, to elicit Nav1.8 currents. **B.** I–V curve of Nav1.8 currents. **C** and **D.** Nav1.8 currents are reduced following incubation with GDF-15 for 10, 20, and 30 min (*P <0.05; **P <0.01, two-way ANOVA). **E.** I–V curves of Nav1.8 currents of vehicle- and GDF-15-treated cells for 20 min (*P <0.05; **P <0.01, two-way RM ANOVA). **F.** Dose-effect curve of GDF-15-induced inhibition of Nav1.8 currents elicited by a single pulse of –10 mV. **G.** Typical traces illustrating the Nav1.8 currents elicited by a single pulse of –10 mV in small-diameter DRG neurons recorded pre- and post-GDF-15 (1.2 nmol/L) incubation for 20 min. **H.** Nav1.8 currents density is decreased by GDF-15 (1.2 nmol/L) incubation for 20 min (*P <0.05. Student’s *t*-test).

Nav1.8 makes a major contribution to the upstroke of APs in small-diameter DRG neurons [43]. Therefore, the modulation of this channel by GDF-15 may be involved in its effect on the excitability of DRG neurons. In the presence of TTX (500 nmol/L), TTX-R Na^+^ currents were recorded in most (approximately 75%) of the small-diameter (<25 μm) DRG neurons. As in our previous report, Nav1.9 currents were inhibited when the membrane potential was held at –60 mV [40]. Nav1.8 currents were recorded in voltage-clamp mode (Fig. 2A). According to the I–V curve of the Nav1.8 channel (Fig. 2B), we selected –10 mV to evoke Nav1.8 currents. To test the effect of GDF-15 on Nav1.8 currents, acutely isolated DRG neurons were incubated with different doses of GDF-15, and Nav1.8 currents were recorded at 10, 20, and 30 min. As shown in Fig. 2C–F, Nav1.8 currents were significantly decreased at 20 and 30 min after 1.2 nmol/L GDF-15. The ED_50_ was calculated to be 1.23 nmol/L. To eliminate the impact of cell size on the amplitude of currents, we also analyzed the current density of Nav1.8 channels. Incubation of GDF-15 (1.2 nmol/L) for 20 min significantly reduced the current density of Nav1.8 channels in DRG neurons either from naïve or arthritic rats (Student’s *t*-test, *t*_(18)_ = 2.91, *P* = 0.01, Fig. 2G, H, S2D, E).

### Effect of GDF-15 on Kinetic Properties of Nav1.8 Channels

**Fig. 3:**
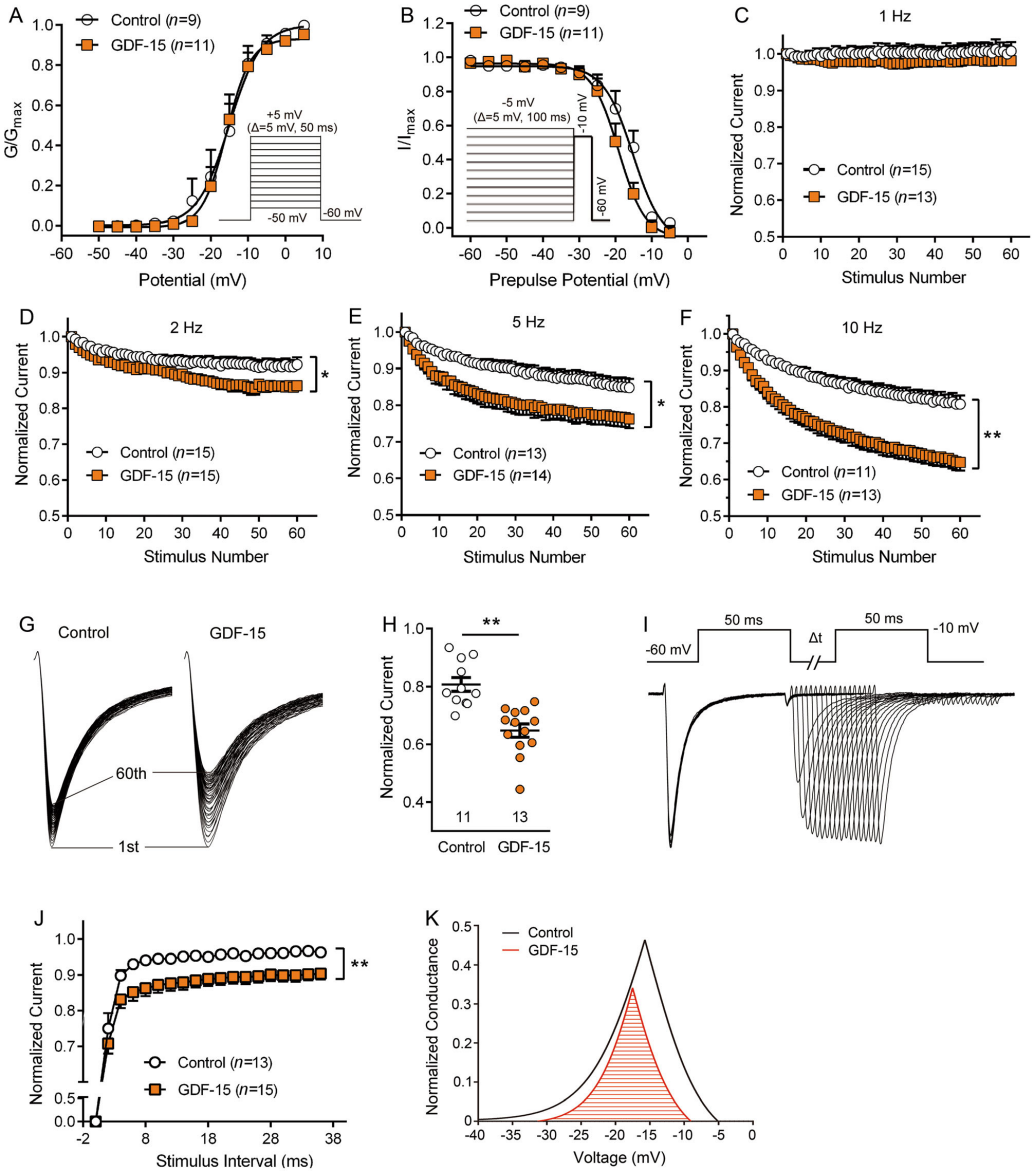
Effects of GDF-15 on the kinetic properties of Nav1.8 channels. **A.** GDF-15 (1.2 nmol/L) does not shift the voltage-dependent activation curve of Nav1.8 channels. **B.** GDF-15 left-shifts the steady-state inactivation curve of Nav1.8 channels in a hyperpolarizing direction. **C–F.** GDF-15 (1.2 nmol/L) exaggerates the frequency-dependent reduction of Nav1.8 currents with increasing stimulation frequency from 1 to 10 Hz (*P <0.05; **P <0.01, two-way RM ANOVA). **G** and** H.** GDF-15 decreases the amplitude of Nav1.8 currents evoked by the 60^th^ pulse of 10 Hz stimulation (**P <0.01, Student’s *t*-test). **I** and **J.** GDF-15 slows the recovery of Nav1.8 channels, using 18 paired-pulse with increasing interstimulus intervals. **K.** GDF-15 reduces the window currents of Nav1.8 channels, according to Boltzmann function fitting.

To further understand the effect of GDF-15 on the gating kinetics of Nav1.8 channels, we analyzed their steady-state activation and steady-state inactivation properties. As shown in Fig. 3, no shift in the voltage-dependent activation curve was seen in the GDF-15-treated group compared with the control (Fig. 3A). The half-maximal activation potentials (*V*_1/2_ activation) were similar in the GDF-15 treatment and control groups (–15.77 ± 0.63 mV *vs* –15.1 ± 1.05 mV, Student’s *t*-test, *t*_(18)_ = 0.57, *P* = 0.5). A voltage protocol (pre-pulse potential: held at –60 mV) differing from the activation curve of Nav1.8 channels, GDF-15 caused a left-shift toward the hyperpolarizing potential of the steady-state inactivation curve (Fig. 3B). The *V*_1/2_ inactivation was –15.36 ± 0.95 mV and –19.11 ± 0.65 mV in the absence and presence of 1.2 nmol/L GDF-15, respectively (Student’s *t*-test, *t*_(18)_ = 3.36, *P* = 0.004). We also analyzed the steady-state activation and inactivation properties of Nav1.8 channels in rats with CFA-induced arthritis and found a left-shifted activation curve compared to naive rats, suggesting that Nav1.8 channels were more sensitive under inflammatory conditions (Fig. S2H). Despite the change in the activation curve of Nav1.8 channels by CFA inflammation, GDF-15 caused a left-shift toward the hyperpolarizing potential of the steady-state inactivation curve but had no influence on the steady-state activation curves in CFA inflammatory rats, similar to naive rats (Fig. S2H, I). The results suggest that GDF-15 exerted its inhibitory effect on Nav1.8 through fast inactivation of the channel.

Previous studies have shown that TTX-resistant Na^+^ channels are highly sensitive to repetitive depolarization [44]. When the interval between stimuli is not enough to restore the channels to the resting state, the number of opening channels will be less than that of the last stimulation. The shorter the interstimulus interval, the slower the recovery of Na^+^ channel from inactivation, which is called frequency-dependent reduction [45]. Consistently, Nav1.8 currents showed a frequency-dependent reduction with increasing frequency of stimulation from 1 to 10 Hz in the vehicle control. In the presence of GDF-15, this frequency-dependent reduction became more pronounced, indicating a use-dependent decay with faster kinetics (Fig. 3C–F). The normalized amplitudes of currents evoked by the 60^th^ pulse at 10 Hz were significantly decreased in the presence of GDF-15 (Fig. 3G, 3H, Student’s *t*-test, *t*_(22)_ = 4.8, *P* <0.0001). We also analyzed the recovery curves of Nav1.8 channels. Channel recovery curves were recorded using 18 paired pulses with increasing interstimulus intervals, and then fitted with an exponential function {*I/I*_max_ *= A*_fast_ [1 *− exp* (*− t/tau*_fast_)] *+ A*_*slow*_ [1 *− exp* (*− t/tau*_slow_)]}. As shown in Fig. 3I, J, GDF-15 significantly slowed the recovery of Nav1.8 channels (two-way RM ANOVA, *F*_(1, 26)_ = 8.85, *P* = 0.006), indicating that GDF-15 slowed the recovery rate of Nav1.8 channels. We also analyzed the window currents of Nav1.8 channels, which represented a small Na^+^ current seen in a “window” of voltages where the activation and steady-state inactivation curves overlapped [46]. When a small fraction of Nav1.8 channels were likely to open at any moment, the larger the overlapping area, and the more opening channels. According to the Boltzmann function fitting, the window currents were reduced in the presence of GDF-15 (Fig. 3K). These data suggest that the inhibition of Nav1.8 currents by GDF-15 may be achieved by accelerating inactivation and slowing recovery of the channel.

### GDF-15 Reduces Nav1.8 Currents via ALK2

**Fig. 4:**
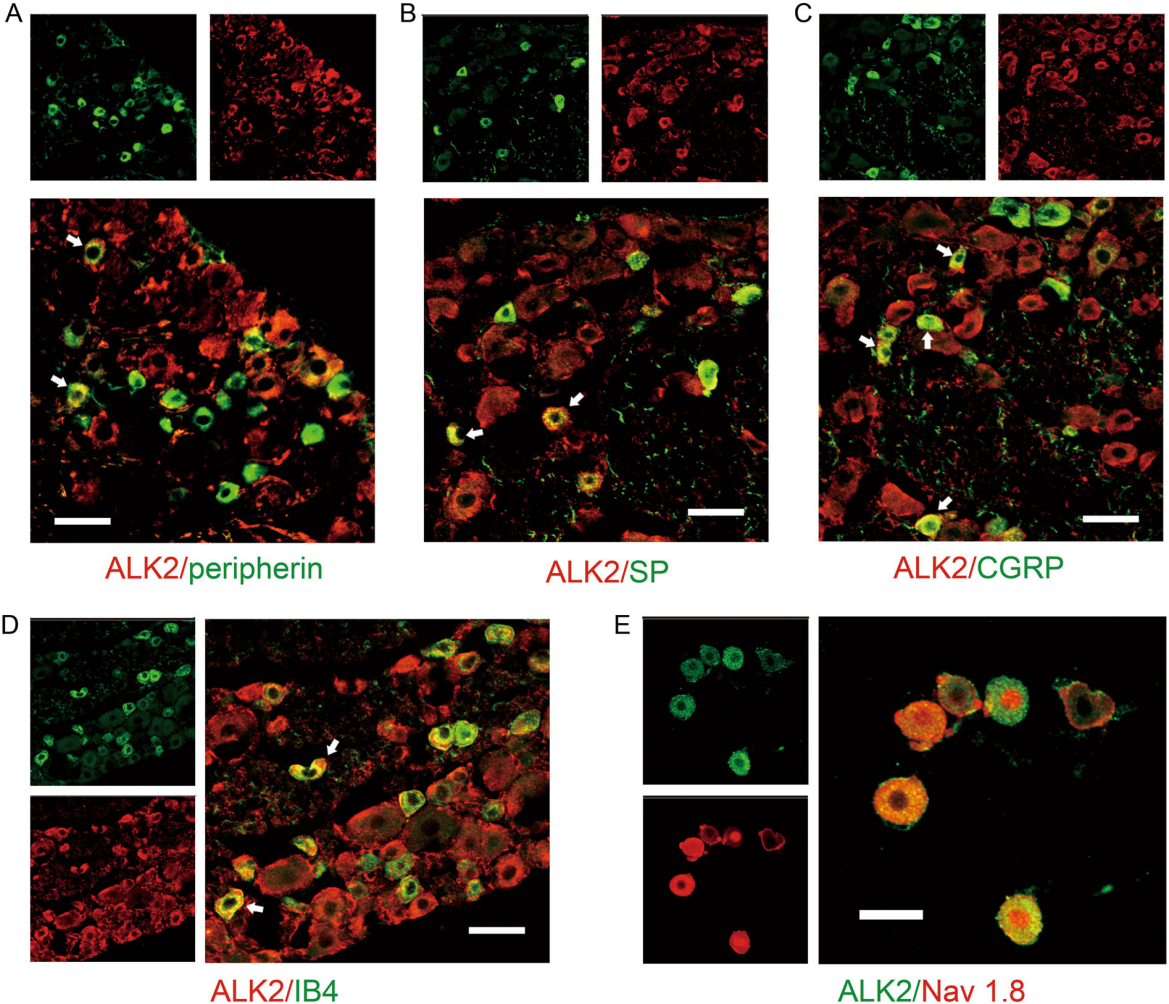
ALK2 expression in DRG neurons. **A–D.** Double immunofluorescence reveals the expression of ALK2 in peripherin- (**A**), substance P- (SP, **B**), calcitonin gene-related peptide- (CGRP, **C**), and isolectin B4- (IB4, **D**) positive neurons in the DRG (scale bars, 50 μm). **E.** Immunocytochemistry double staining of ALK2 and Nav1.8 in isolated DRG neurons (scale bar, 30 μm).

**Fig. 5:**
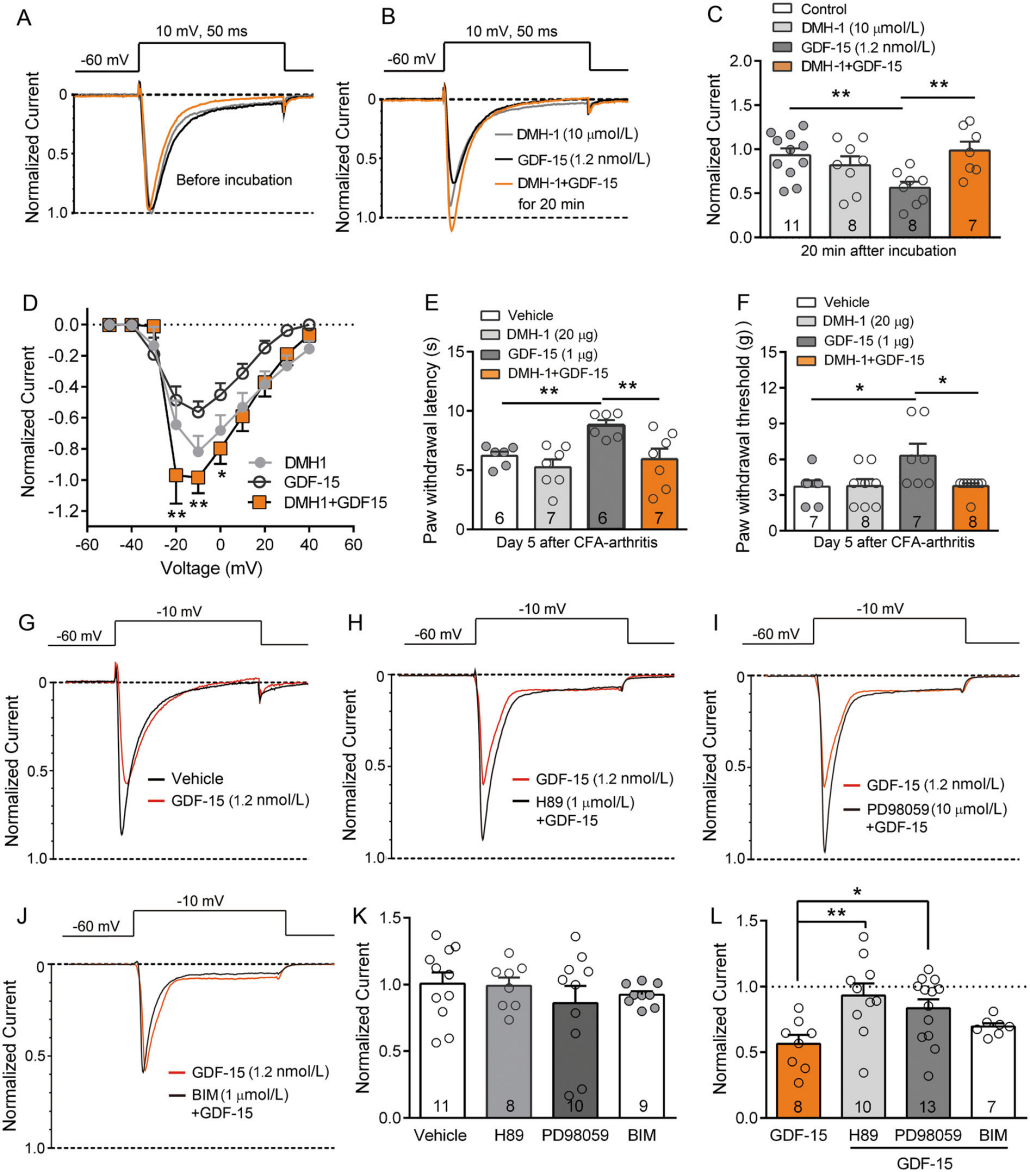
GDF-15 reduces Nav1.8 currents *via* ALK2 and intracellular signaling of PKA and ERK in small-diameter DRG neurons. **A–C.** The ALK2-specific inhibitor DMH-1 blocks GDF-15-induced inhibition of Nav1.8 currents. DMH-1 *per se* does not affect Nav1.8 currents (**P <0.01, one-way ANOVA). **D.** I–V curves of Nav1.8 currents of cells treated with DMH-1, GDF-15, and DMH-1+GDF-15 for 20 min. **E** and **F.** Peripheral DMH-1 blocks GDF-15-induced inhibition of thermal hyperalgesia (**E**) and mechanical allodynia (**F**) in CFA-arthritic rats. (*P <0.05, **P <0.01, one-way ANOVA). **G–J.** Typical traces illustrating that the PKA inhibitor H89 (**H**) and MEK/ERK inhibitor PD98059 (**I**), but not the PKC inhibitor BIM-1 (**J**) block the GDF-15-induced inhibition of Nav1.8 currents elicited by a single pulse of –10 mV in DRG neurons with GDF-15 (1.2 nmol/L) incubation for 20 min. **K.** H89, PD98059, or BIM per se do not change the Nav1.8 currents. **L.** Inhibition of Nav1.8 currents by GDF-15 is blocked by H89 and PD98059, but not BIM (*P <0.05; **P <0.01, one-way ANOVA).

ALK2, encoded by the ACVR1 gene, is a BMP type-I receptor subtype that mediates the actions of multiple BMP/TGF-β superfamily molecules [47, 48]. Double immunostaining showed that ALK2 was widely distributed in DRG neurons, including medium-, small-diameter nociceptive (peripherin-positive), and large-diameter (peripherin-negative) neurons (Fig. 4A). Both peptidergic (SP- and CGRP-positive) and non-peptidergic (IB4-positive) neurons expressed ALK2 (Fig. 4B–D). ALK2 was also co-localized with Nav1.8 channels in DRG neurons (Fig. 4E). To address whether the GDF-15-induced attenuation of Nav1.8 currents was mediated by ALK2, we examined the effect of dorsomorphin homolog 1 (DMH-1), an ALK2-specific inhibitor of the inhibitory effects of GDF-15 on Nav 1.8 currents. Pretreatment of DRG neurons with 10 μmol/L DMH-1 significantly blocked the GDF-15-induced suppression of Nav 1.8 currents (Fig. 5A–D, one-way ANOVA, *F*_(3, 30)_ = 4.5, *P* = 0.01). We also tested the effects of DMH-1 on GDF-15-induced analgesia in CFA arthritic rats. As shown in Fig. 5E, 5F, intraplantar injection of DMH-1 (20 μg/50 μL) completely blocked the analgesic effect of GDF-15 (one-way ANOVA, *F*_(3, 26)_ = 5.5, *P* = 0.006 for PWL; *F*_(3, 26)_ = 3.9, *P* = 0.02 for PWT).

### PKA and ERK Signaling Participate in GDF-15-induced Inhibition of Nav1.8 Channels

Several non-Smad signaling pathways have been linked to rapid responses of multiple BMP/TGF-β superfamily molecules. PKA, PKC, and ERK have been implicated in regulating the activity of Nav1.8 currents in DRG neurons [40, 49–51]. We, therefore, assessed whether PKA, PKC, or MEK/ERK were involved in the inhibition of Nav1.8 channels by GDF-15. Although the PKA inhibitor H89 (1 μmol/L) and the MEK/ERK inhibitor PD98059 (10 μmol/L) did not affect Nav1.8 currents *per se*, either of them significantly blocked the GDF-15-induced suppression of Nav1.8 currents (Fig. 5G–L, one-way ANOVA, *F*_(3,34)_ = 4.4, *P* = 0.01). In contrast, the PKC inhibitor BIM (1 μmol/L) did not block the inhibition of Nav1.8 currents by GDF-15 (Fig. 5J–L).

## Discussion

Previously, the BMP/TGF-β superfamily members TGF-β1, Activins, and BMPs have been involved in chronic pain, including neuropathic, inflammatory, and cancer pain [18, 20, 52]. In the present study, we further provided insight into the analgesic effect of GDF-15, another member of the BMP/TGF-β superfamily, at the peripheral level. Our results showed that peripheral application of GDF-15 significantly suppressed the behavioral responses to noxious thermal and mechanical stimuli in normal and CFA-arthritic rats. We also showed that GDF-15 dose-dependently reduced Nav1.8 currents and decreased the excitability of small-diameter sensory neurons. This inhibition of Nav1.8 currents was blocked by a selective inhibitor of ALK2, suggesting that ALK2 is directly involved in GDF-15-induced changes in Nav1.8 activity. In addition, PKA and MEK/ERK signaling are responsible for the activation of Nav1.8 currents in response to GDF-15 in DRG neurons. These results suggest a peripheral mechanism of GDF-15 analgesia.

Among the pore-forming α subunits of Nav channels, Nav1.3, Nav1.6, Nav1.7, Nav1.8, and Nav1.9 Na^+^ channels are present in DRG neurons and contribute to somatosensory signal transmission [25]. In particular, TTX-R Nav1.8 is expressed only in a subset of sensory neurons of which >85% are nociceptors [24, 53]. Accumulating evidence points out that the Nav1.8 channel plays an important role in peripheral pain processing [54, 55]. Nociceptive signals evoke a dynamic change of Nav1.8 channels in the DRG: for example, paclitaxel-induced neuropathy [56], chronic compression of the DRG [26, 57], spinal nerve ligation, or local inflammation by the application of formalin, carrageenan, or CFA [58]. Either the physiological or pathological pain is alleviated by Nav1.8 sodium channel blockers [58–60] or Nav1.8 knockout [33, 54]. Human genetic evidence suggests that gain-of-function mutations in Nav1.8 channels contribute to painful peripheral neuropathy [29, 34].

Nav channels are necessary for the generation and conduction of APs, and Nav1.8 channels have been shown to contribute to the upstroke of the AP and support high repetitive firing rates [43]. Our data showed that GDF-15 significantly increased the AP threshold and decreased the frequency of AP discharges in small-diameter DRG neurons, suggesting that the decreased excitability by GDF-15 may be related to the regulation of Nav1.8 channels. Indeed, GDF-15 dose-dependently suppressed Nav1.8 currents and reduced window currents. Moreover, GDF-15 prompted a prominent hyperpolarizing shift in the inactivation but did not affect the activation. The frequency-dependent reduction was more pronounced by GDF-15, suggesting a significantly delayed recovery effect on Na^+^ channels. This profile is quite consistent with the possibility that GDF-15 selectively affects the inactivated Nav1.8 channels.

The level of GDF-15 in the serum of rheumatic arthritis patients is higher than in healthy people [41, 42]. Exogenous GDF-15 decreases NF-κB and downregulates interleukin-8 [13], suggesting the involvement of GDF-15 in the inflammatory disease process. In the current study, we further demonstrated that peripheral GDF-15 significantly suppressed nociceptive responses and relieved the mechanical allodynia and thermal hyperalgesia in normal and CFA-arthritic rats, corresponding to the inhibitory effect of GDF-15 on the excitability of small-diameter DRG nociceptor neurons. Consistently, some other members of the BMP/TGF-β superfamily, such as activins, BMPs, and TGF-βs, have been shown to modulate nociceptive information [16–18, 52]. The inhibitory effects GDF-15 on nociceptive behaviors and Nav1.8 currents were markedly blocked by DMH-1, a specific inhibitor of ALK2, suggesting that ALK2 mediates the action of GDF-15. In support, ALK2 was widely expressed in DGR neurons, especially medium- and small-diameter nociceptive neurons, and co-localized with Nav1.8 channels.

**Fig. 6:**
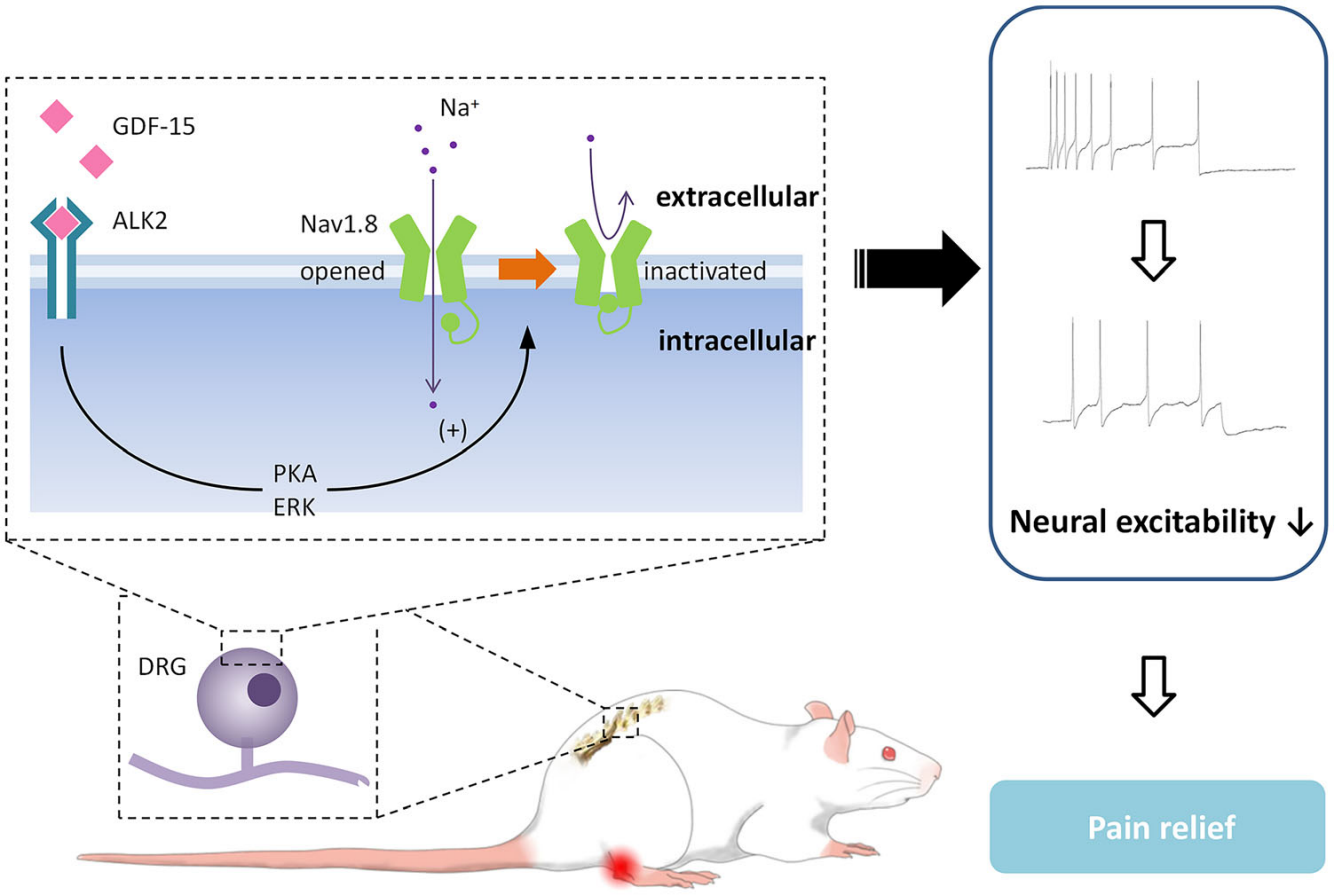
Schematic showing how GDF-15 modulates peripheral nociceptive information. GDF-15 inhibits Nav1.8 on nociceptors by membrane ALK2 and downstream intracellular signals, such as PKA and ERK, leading to a reduction in the excitability of DRG neurons and pain relief.

ALK2 is a BMP type-I receptor subtype and plays an important role in the development of bones, muscles, brain, and other organs. ALK2 interacts with type II receptors to form transmembrane heterotetrameric receptor complexes and mediates the actions of multiple BMP/TGF-β superfamily molecules [47, 48]. Multiple intracellular signaling pathways are associated with the rapid actions of the TGF-β/BMP superfamily. It has been reported that GDF-15 activates BMP receptors and PI3K/Akt/mTOR and ERK signaling to rapidly regulate K^+^ channels and Ca^2+^ channels [38, 39, 61]. In this study, we revealed that inhibition of PKA and ERK prevented GDF-15 from suppressing Nav1.8 currents. In support of this, it has also been reported that PKA [40, 62] and ERK [63, 64] modulate the Nav1.8 currents in small-diameter DRG neurons (Fig. 6).

Despite the fact that chronic pain is unevenly distributed between the sexes, occurring more frequently in women, and that clinical trials must incorporate women into the trial design [65, 66], current basic science research focuses primarily on male subjects. A recent study showed that patients with high GDF-15 levels have lower testosterone levels [67], suggesting an effect of male hormones on endogenous GDF-15 levels. Our findings demonstrated an analgesic effect of peripheral GDF-15 in female rats. Thus, future studies are warranted to investigate GDF-15 and its receptors signaling not only in different pain models but also in different sexes.

In summary, we found in the present study that GDF-15 dose-dependently suppressed Nav1.8 currents, aggravated the use-dependent reduction of Nav1.8 channels, attenuated the excitability of DRG neurons, and thus rapidly and effectively relieved pain. These findings provide an insight into the mechanisms underlying the analgesia of peripheral GDF-15. Despite the importance of the DRG, as a gatekeeper for the primary afferent nerves in acute and chronic pain development, the evidence for current therapeutic strategies is poor. This study might provide a potential neural target for an attempt to manage pain.

## Supplementary Information

Below is the link to the electronic supplementary material.

Supplementary file1 (PDF 506 kb)
