# Supplementary material for: Growth Differentiation Factor-15 Produces Analgesia by Inhibiting Tetrodotoxin-Resistant Nav1.8 Sodium Channel Activity in Rat Primary Sensory Neurons
Source: Neurosci Bull. 2021 Jun 2;37(9):1289–302. doi: 10.1007/s12264-021-00709-5 (PMC8423960; doi:10.1007/s12264-021-00709-5)
Supplement: Supplementary file 1 — Supplementary file1 (PDF 506 kb) [file 12264_2021_709_MOESM1_ESM.pdf]

Supplemental Figures and Figure Legends

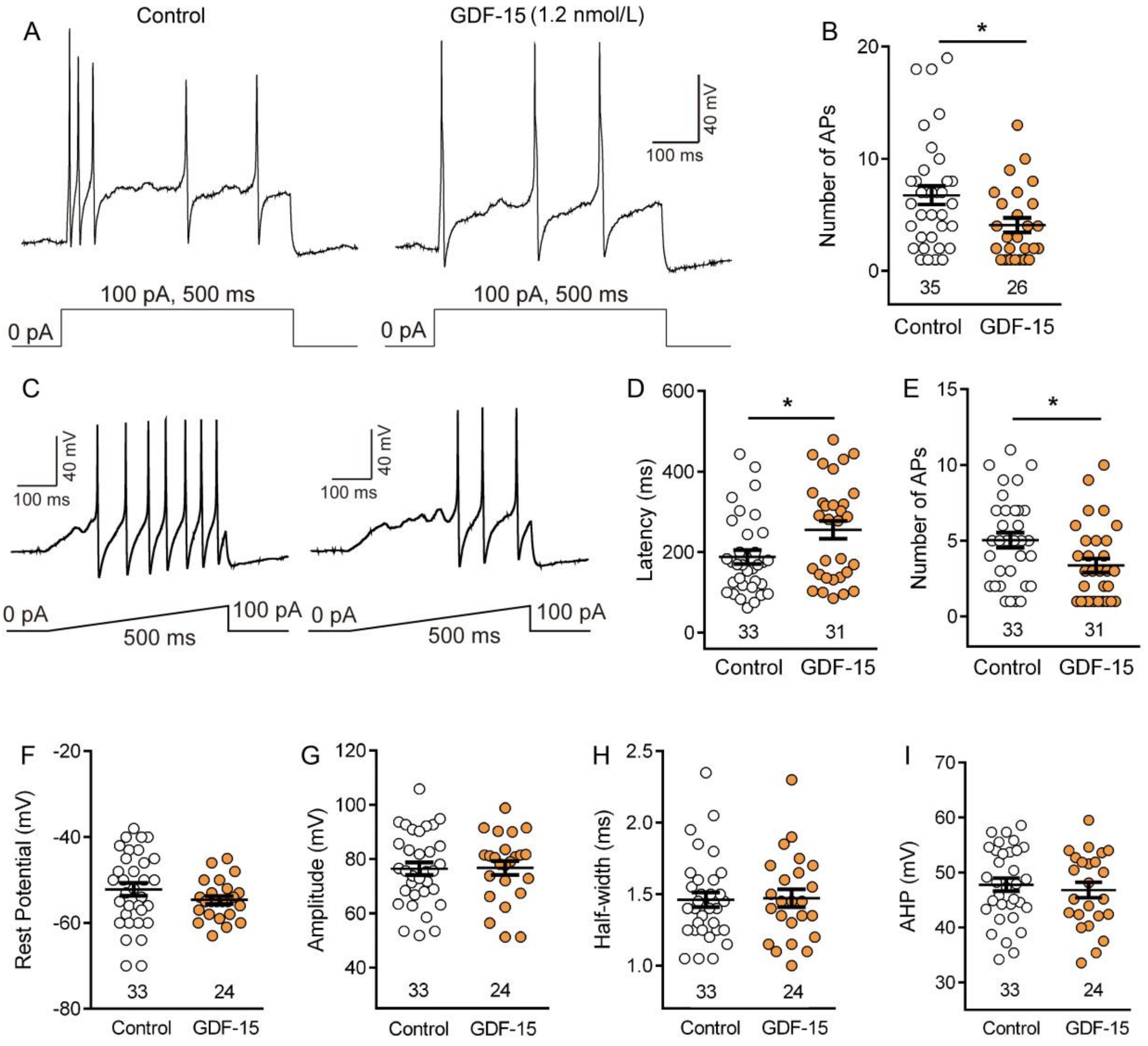

Fig. S1 Effects of GDF-15 on APs and membrane properties of DRG neurons. **A.** The AP firing response to a 100-pA depolarizing current pulse in control and GDF-15-treated DRG neurons. **B.** Summary data showing the inhibitory effects of GDF-15 on the firing rate in DRG neurons (*P <0.05, Student's t-test). **C.** The AP firing response to a ramp current stimulation (0–100 pA, 500 ms) in control and GDF-15-treated DRG neurons. **D and E.** GDF-15 prolongs the latency to the first AP (**D**) and reduces the number of APs (**E**) (*P <0.05, Student's t-test). **F–I.** The effects of GDF-15 on resting membrane potential (**F**), AP amplitude, AP half-width, and after-hyperpolarization potential (AHP) (Student's t-test).

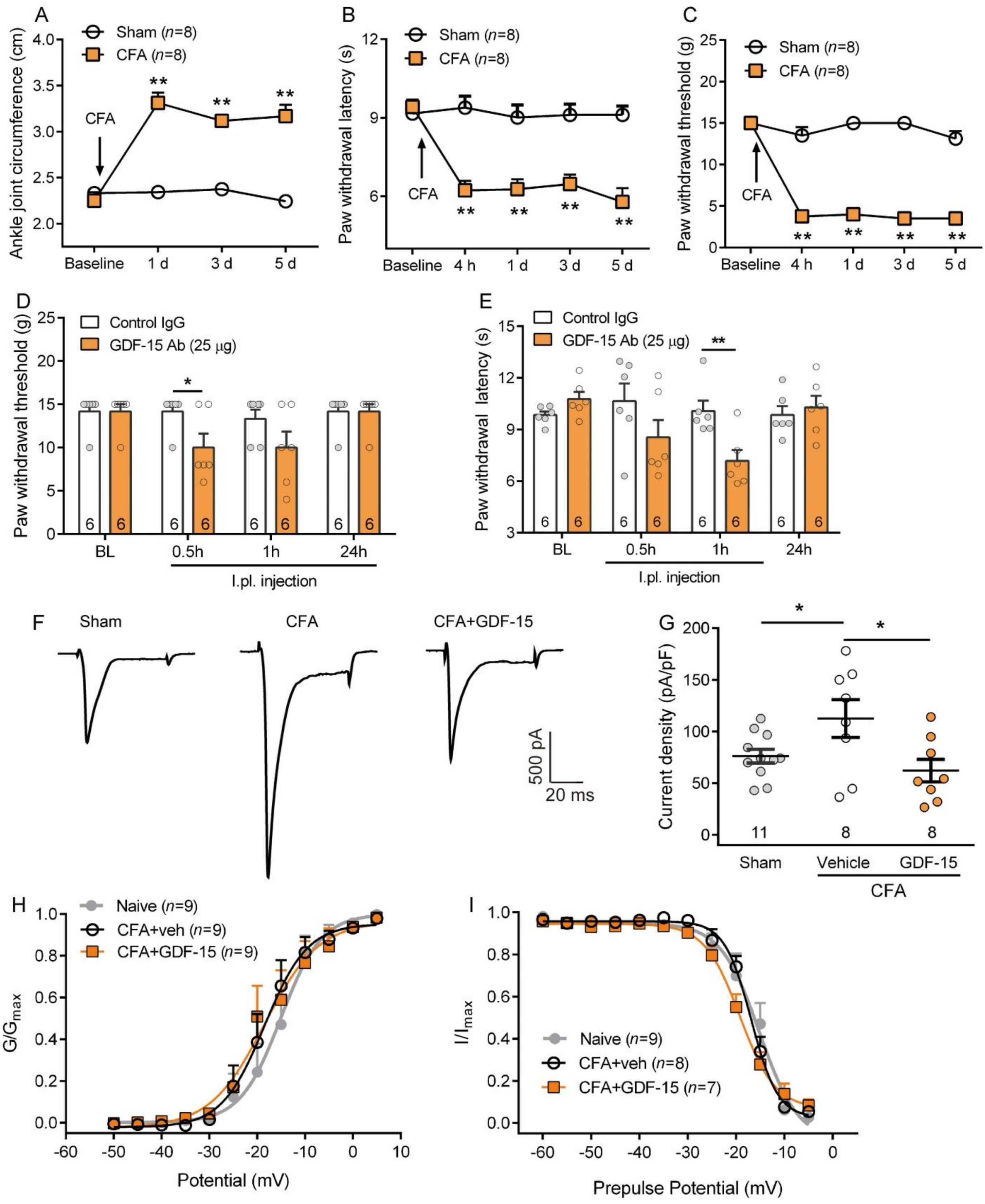

Fig. S2 Effects of GDF-15 on Nav1.8 channels of DRG neurons from CFA-arthritic rats. **A–C.** Intra-ankle articular injection of CFA increases the circumference of the ankle joint (**A**), and produces thermal hyperalgesia (**B**) and mechanical allodynia (**C**) (***P* <0.01, two-way RM ANOVA). **D and E.** Intraplantar injection of GDF-15 antibody (GDF-15 Ab, 25 μg) to block the endogenous GDF-15

results in a decreased mechanical (**D**) and thermal (**E**) response threshold (**P* <0.05, ***P* <0.01, two-way RM ANOVA). **F.** Examples showing the Nav1.8 currents of DRG neurons from sham and CFA-arthritic rats. **G.** GDF-15 reduces the CFA-induced increase in Nav1.8 current density on day 5 after CFA (**P* <0.05, one-way ANOVA). **H and I.** CFA-inflammation induces a left-shifted activation curve of Nav 1.8 channels (**H**), but does not change the steady-state inactivation curve (**I**). GDF-15 (1.2 nmol/L) does not affect the steady-state activation curves of Nav1.8 channels (**H**), but causes a left-shift toward hyperpolarizing potential of the steady-state inactivation curve (**I**).
